# Prevalence and influences of preschoolers’ sedentary behaviors in early learning centers: a cross-sectional study

**DOI:** 10.1186/s12887-015-0441-5

**Published:** 2015-09-18

**Authors:** Patricia Tucker, Leigh M. Vanderloo, Shauna M. Burke, Jennifer D. Irwin, Andrew M. Johnson

**Affiliations:** School of Occupational Therapy, Faculty of Health Sciences, University of Western Ontario, London, Ontario N6G 1H1 Canada; Health and Rehabilitation Sciences, Faculty of Health Sciences, University of Western Ontario, London, Ontario N6G 1H1 Canada; School of Health Studies, Faculty of Health Sciences, University of Western Ontario, London, Ontario N6A 5B9 Canada

**Keywords:** Sedentary time, Preschoolers, Early learning facilities, Accelerometers

## Abstract

**Background:**

Recent research has highlighted the need for increased evidence regarding the sedentary activity levels of preschoolers. Given the large proportion of time this population spends in various early learning facilities, the exploration of sedentary behaviors within this particular environment should be a priority. The purpose of the study was two-fold: (1) to compare sedentary time of preschoolers in three different early learning environments (i.e., full-day kindergarten [FDK], center-, and home-based childcare); and (2) to assess which characteristics (i.e., staff behaviors, sedentary environment, fixed play environment, portable play environment, sedentary opportunities) of these early learning environments influence preschoolers’ sedentary time.

**Methods:**

Data collection occurred between September 2011 and June 2012. Preschoolers’ sedentary time was measured using Actical™ accelerometers at a 15 s epoch. The Environment and Policy Assessment and Observation (EPAO) tool was used to assess the sedentary environment of participating early learning classrooms, and those subscales (*n* = 5) that were evidence-informed as potentially influencing sedentary time in early learning centers were explored in the current study. A linear mixed model ANCOVA was carried out to determine the differences in sedentary time based on type of early learning environment while direct entry regression analyses were performed to describe the relationships between sedentary time and the five sedentary-specific EPAO subscale.

**Results:**

Preschoolers (*n* = 218) from 28 early learning programs (i.e., 8 FDK, 9 centre-, and 8 home-based childcare facilities) participated. Accelerometry data revealed that preschoolers attending centre-based childcare engaged in the highest rate of sedentary time (41.62 mins/hr, *SD* = 3.78) compared to preschoolers in home-based childcare (40.72 mins/hr, *SD* = 6.34) and FDK (39.68 mins/hr, *SD* = 3.43). The models for FDK, center-based childcare, and home-based childcare, comprised each of the five EPAO subscales accounted for 10.5 %, 5.9 %, and 40.78 % of the variability in sedentary time, respectively. Only the models for FDK and home-based childcare were found to be statistically significant (*p* < .05).

**Conclusions:**

This is the first exploration of differences in sedentary time among preschoolers in different early learning arrangements. Findings highlight the substantial portion of the day preschoolers spend in sedentary pursuits, and subsequently, the ongoing need to reduce preschoolers’ sedentary time in early learning programs, particularly among those attending centre-based childcare facilities.

## Background

Sedentary behaviors have received recent attention in light of the negative consequences associated with these activities [1–4]. Specific to preschoolers (i.e., children aged 2.5–5 years), high participation in sedentary behaviors (i.e., screen viewing and prolonged periods of sitting) have been associated with a variety of negative health consequences including higher skinfold measurements [1] and body mass index (BMI) [2] during childhood. A recent review by LeBlanc et al. revealed that increased screen time (a commonly used proxy for sedentary time) was associated with increased adiposity and negative outcomes in psychosocial health and cognitive development among this cohort [3]. Moreover, excessive screen-viewing has been linked to prevalent feelings of boredom and sadness [5], and issues with sleep [6]. As such, researchers have begun to explore sedentary time as a unique construct, rather than merely the opposite of physical activity. Specifically, many researchers have postulated, based on recent evidence, that sitting too much and exercising too little are separate and distinct risk factors for children and adults risk for chronic disease [4, 7, 8].

The importance of considering sedentary time as a unique construct has become increasingly apparent as a recent report by Colley et al. revealed that preschoolers engaged in sufficient levels of physical activity, but were still highly sedentary over the course of a day [9]. In fact, a number of accelerometer-based studies have demonstrated high levels of sedentary time among young children [10–12]. The Canadian sedentary behavior guidelines for the preschool population recommend children aged 3–4 years engage in less than 1 hour per day of screen time, and those 5 years of age engage in less than 2 hours per day [13]. While the focus of the guidelines is screen-viewing rather than sedentary time as a whole, these guidelines provide a benchmark against which parents can gauge their children’s behaviors. When considering this recommendation, Canadian data have suggested that most preschoolers are not achieving this goal [14]. Moreover, findings from a recent systematic review showed that 6 out of 8 studies that reported on screen-viewing behaviors among preschoolers exceeded the 1-hour guideline [15]. This finding is troublesome given that this review focused solely on screen-viewing activity within the childcare environment, thus permitting the possibility for additional screen time outside of childcare hours. Further, Colley et al. revealed that in a representative sample of Canadian preschoolers, participants were, on average, spending approximately 50 % of their day (nearly 6 hours) in sedentary pursuits [9].

Temple et al. and Vanderloo et al. have provided evidence regarding the high levels of sedentary time occurring in early learning facilities within Canada [11, 12]. Specifically, Temple’s group explored home-based childcare facilities and reported that preschoolers spent an average of 39.49 mins/hr in sedentary behaviors [12]. Likewise, Vanderloo et al. reported an average of 40.64 mins/hr of sedentary time among preschoolers in center-based childcare facilities [11]. The high levels of sedentary behaviors have been confirmed internationally [10, 16] and are disconcerting given the large number of preschoolers that attend childcare [17]. While previous studies have explored sedentary time in home- and center-based childcare independently, no study to date has measured sedentary time across multiple childcare environments or explored the specific characteristics of these environments that are correlated with sedentary time (which is important given they are different environments, with different resources and regulations). In light of the high sedentary time captured by Temple et al. and Vanderloo et al. [11, 12], intervention is warranted to reduce preschoolers’ sedentary time in early learning facilities. However, to ensure interventions are evidence-informed and appropriately designed, Hinkley et al. have argued that an understanding of the factors that influence this behavior, inclusive of identifying modifiable correlates, is warranted [18]. Individual studies have identified components of the early learning environment that may be important to target. For example, Sugiyama et al. found that children in centers with lower staff-child ratios (six or less children per staff member) and those that used indoor space for gross motor activities (rather than relying solely on outdoor time) engaged in less sedentary time [19]. Vanderloo et al. provided further support for this finding as they identified that preschoolers in center-based childcare engaged in significantly more sedentary time indoors than outdoors [11]. The play equipment available within early learning environments (i.e., fixed and portable) has also been identified as an influential factor with regard to the activity behaviors of preschoolers. Specifically, preschoolers have been reported to be more sedentary when a greater number of fixed equipment structures are available [11, 12]. Possible explanations for this may be the ‘standing around’ that occurs while waiting to use the fixed equipment, or that childcare staff discourage running on these pieces to reduce safety concerns.

While Colley et al.’s recent work provides a starting point for understanding Canadian preschoolers’ sedentary time [9], the high levels apparent in early learning facilities is discouraging. Recent research has highlighted the need for increased evidence regarding the physical activity and sedentary time of preschoolers [18], and argues that exploring these behaviors within the early learning environment should be a priority [20]. More specifically, Colley et al. stressed that a much-needed area of future research include an examination of the influence of enrolment in childcare programs on activity levels [9]. As such, the purpose of this study was two-fold: 1. to compare preschoolers’ sedentary time in three different early learning environments (i.e., full-day kindergarten [FDK], center-, and home-based childcare); and 2. to assess which modifiable correlates (i.e., staff behaviors, sedentary environment, sedentary opportunities, portable play environment, fixed play environment) of *each* of these early learning environments influence preschoolers’ sedentary time. For the purpose of this study, center-based childcare referred to any formalized setting which provides licensed childcare to a large number of preschoolers (approximately 16) on a full- or part-time basis [21]. Home-based childcare typically involves a smaller number of children (usually no more than 5 plus the provider’s own children) across various age groups (e.g., 1–11 years), in a home environment and can operate in a licensed or unlicensed capacity [12]. Finally, FDK programming requires children to attend all day, every week day (i.e., Monday to Friday from approximately 9 am to 3 pm), and receive instruction from both a teacher and an early childhood educator. More information about the various settings has been published elsewhere [21, 30]. Because some home-based childcare facilities are not licensed, it was hypothesized that preschoolers attending home-based childcare would engage in higher levels of sedentary behaviors.

## Methods

### Study design

This research was conducted as part of the larger Learning Environments’ Activity Potential for Preschoolers (LEAPP) study (a detailed account of the methodology has been provided elsewhere [21]). Study procedures and materials were pilot tested by the research team [11] and data collection took place between September 2011 and June 2012 in London, Ontario, Canada. This 2-year cross-sectional study, along with all related materials, received ethical approval from the Office of the Research Ethics Board at the University of Western Ontario in Canada.

### Participants

Preschool children aged 2.5–5 years from three different early learning environments (i.e., FDK, center-, and home-based childcare) were invited to participate. Early learning environments were contacted by the project coordinator and invited to participate. Recruitment efforts were targeted for each early learning arrangement; originally all FDK and home-based childcare facilities were invited to participate. Because a random sample of FDK classrooms or home-based childcare was not possible (because of the lower number of facilities), purposeful sampling was undertaken to recruit geographically diverse centre-based childcare facilities. For a detailed account of the recruitment protocol, see Tucker et al. [21]. For those environments that had more than one classroom able to participate (i.e., FDK and centre-based childcare), both were invited to participate or one was selected at the discretion of the director/principal. Parents/guardians of preschoolers were then provided with a letter of information detailing the study, along with a corresponding consent form to sign if interested in participating in the study. Only children who received parental/guardian consent were eligible to participate.

### Tools

To measure time spent in sedentary behaviors, Actical™ (MiniMitter, Bend, Oregon) accelerometers were worn by preschoolers for 5 consecutive days during childcare hours only. The accelerometers were placed on the right hip of each child, and early learning staff were asked to record the ‘on’ and ‘off’ times of the device for each child. A 15-second epoch length was used, consistent with previous research [11, 12]. Current evidence supports the appropriateness of using accelerometers to measure sedentary time as it provides an objective and accurate depiction of minutes spent being inactive [22]. While this device lacks contextual information regarding the types of sedentary behaviors in which these children were engaging (e.g., television viewing, computer time, reading, etc.), this information was captured via the Environment and Policy Assessment and Observation’s (EPAO) Sedentary Opportunities Subscale.

The EPAO tool [23–25], created to examine the physical activity and sedentary behaviors environment in center-based childcare, was used. Two independent research assistants completed the EPAO on a weekday during childcare hours. These research assistants were trained by the primary investigator on the use and administration of the EPAO tool, including discussing the tool’s completion instructions and associated protocol. One of the research assistants also pilot tested the EPAO in a previous study, so had in-depth knowledge of the tool. While all scales were collected, for the purpose of the current research objectives, and consistent with past research [26], only those subscales (*n* = 5) which were evidence-informed as potentially influencing sedentary time in early learning centers were explored in the current study. Two EPAO subscales – Sedentary Environment and Sedentary Opportunities – examined the sedentary environment (e.g., availability of screens) and the opportunities (e.g., sitting time within the curriculum) for inactive behavior within this setting. Additionally, the Staff Behaviors, Fixed Play Environment, and Portable Play Environment subscales were used as previous research has suggested that childcare staff influence the activity behaviors of preschoolers, and the types of equipment present in the childcare facility may be correlated to sedentary activity [11, 26]. Consequently, these additional subscales may shed important insight into what factors influence sedentary behaviors in early learning environments. During the week of data collection, two research assistants entered the early learning facilities and examined the environment present in each for one full day. Please see Tucker et al. for a full methodological account of this process [21].

A demographic questionnaire was also administered to parents/guardians of preschoolers. Such items included: child’s ethnicity, child’s enrollment status in an early learning program (i.e., full-time vs. part-time), family arrangement, parent/guardian education levels, annual household income, and, parental/guardian role modeling.

## Statistical analysis

Accelerometer data was downloaded and *KineSoft* version 3.3.62 (KineSoft, Saskatchewan, Canada) software was used to apply quality control measures to the data; non-wear time was defined as 60 minutes of consecutive zeroes (which accounted for nap time, where applicable) [27], and participants with 3 or more valid days were included in all analyses (where a valid day was defined as a minimum of 5 hours of accelerometer wear-time [28]). Based on these parameters, 73 % of participants had sufficient data (*n* = 218). Using Pfeiffer et al.’s cut-points for the preschool cohort (i.e., < 50 counts per 15 second epoch; functionally equivalent to sitting) [29], average daily sedentary time was calculated by dividing the total sum of minutes of sedentary behaviors on valid days by the number of valid days. In line with previous research [11], sedentary time per hour of wear time was calculated to account for preschoolers’ varying attendance length within their respective early learning facility.

Means and standard deviations were calculated to examine participants’ demographic characteristics. To account for the clustered data structure and to examine the study’s primary outcome measures, a linear mixed model ANCOVA was carried out to determine the differences in sedentary time based on type of early learning environment. An ANCOVA was appropriate because it allowed us to test if there was a difference in sedentary time between the three groups of preschoolers (i.e., early learning environments) while accounting for sex and early learning environment. The early learning centers were entered as *strata* and individual classrooms (within these centers) as *clusters* for the purpose of the present paper’s analysis. Unstandardized residual scores were calculated by running a regression analysis of age onto sedentary time in order to account for the effect of age. These residual scores were used in subsequent ANCOVA analyses. The main effects and interaction for the following fixed factors were included in the model: type of early learning environment (i.e., FDK, center-based childcare, home-based childcare) and sex (i.e., boy, girl). Random effects included classrooms clustered within early learning facilities. Tukey’s HSD was used to examine the post-hoc comparisons of where the differences in sedentary time existed across the three types of early learning environments.

To examine the influential characteristics of the early learning environments, the EPAO scoring tool was used to tally the results of the five applicable subscales [24]. Each subscale score ranged from 0 to 20, with a lower score representing a more conducive environment for sedentary behaviors specific to the Staff Behaviors, Fixed Play Environment, and Portable Play Environment subscales. For the Sedentary Environment and Sedentary Opportunities subscales, a higher score out of 20 indicated a more sedentary environment. Two independent observers coded the EPAO subscales and intraclass correlation coefficients (ICCs) were calculated. All ICCs were computed using an absolute agreement definition. An ICC was not calculated for one subscale (i.e., Sedentary Environment), as it had a perfect correlation on the composite scores between the two reviewers. The inter-rater reliability for the remaining four subscales are presented elsewhere [30]. Because all subscales represent composite scores, an average ICC score was used. Direct entry regression analyses were performed to describe the relationships between sedentary time and the five sedentary-specific EPAO subscales. By examining the adjusted *R*^2^ values for each model, the coefficients of determination (*R*^2^) were ascertained.

## Results

A total of 8 (response rate = 57 %) FDK schools (*n* = 149 preschoolers), 9 (response rate = 30 %) center-based childcare facilities (*n* = 117 preschoolers), and 11 (response rate = 11 %) home-based childcare facilities (*n* = 31 preschoolers) participated in the current study, for response rates of 29 %, 50 %, and 93 % for preschoolers, respectively. Only those children with sufficient activity data (i.e., those who wore the accelerometer for 3 days with 5 hours or more each day) were included in the present analyses (*n* = 218). The mean age of the preschool participants was 4.18 years (*SD* = 0.97; 53.2 % female). Average daily accelerometry wear time was 406.21 minutes (*SD* = 53.75). Home- and center-based childcare facilities required nap times for the preschoolers; average daily ‘quiet time’ was 73.17 minutes (*SD* = 44.29). Children attending FDK did not take naps. See Table 1 for complete preschooler participant demographic information.

**Table 1:** Overall Preschooler and Family Demographic Information (*n* = 218), and Demographics by Early Learning Environment

	Overall	Centre-Based Childcare	Home-Based Childcare	FDK
Sex				
Male	102	32	9	62
Female	116	39	12	65
Early learning environment				
Home-based childcare	20	---	---	---
Center-based childcare	71	---	---	---
Full-day kindergarten	127	---	---	---
School/childcare status				
Part-time	23	16	6	1
Full-time	193	55	14	124
Preschooler’s ethnicity				
Caucasian	176	57	19	101
African Canadian	1	0	0	1.0
Aboriginal	2	0	0	2.0
Arab	5	2.0	0	3.0
Latin American	2	2.0	0	0
Asian	10	4.0	0	6.0
Other	12	4.0	0	8.0
Highest level of parent/guardian education				
High School	32	12	2	18
College	68	17	12	39
University	66	22	6	38
Graduate School	44	18	1	25
Approximate yearly household income				
Less than $20,000	14	4	1	9
$20,000–$39,999	17	11	0	6
$40,000–$59,999	20	8	0	12
$60,000–$79,999	19	6	4	9
$80,000–$99,999	28	5	5	18
$100,000–$119,999	23	11	2	10
More than $120,000	48	10	2	36

*Note.* All values shown may not add up to *n* = 218 as some individuals chose not to answer certain questions

### Preschoolers’ sedentary time across the different early learning environments

Preschoolers engaged in high levels of sedentary time. Specifically, preschoolers attending center-based childcare engaged in the highest rates of sedentary time (41.62 mins/hr, *SD* = 3.78), followed by preschoolers in home-based childcare (40.72 mins/hr, *SD* = 6.34) and FDK (39.68 mins/hr, *SD* = 3.43). Significant differences in sedentary time were observed between FDK and centre-based childcare (*p* < .05), with preschoolers in center-based childcare engaging in significantly more sedentary time than preschoolers attending FDK.

### EPAO subscales and sedentary time

Three direct entry linear regression models were fit, one each for FDK, center-based childcare, and home-based childcare. In each model, the five EPAO subscales (i.e., Sedentary Opportunities; Sedentary Environment; Fixed Play Environment; Portable Play Environments; and Staff Behaviors) were used to predict sedentary time. These models accounted for 10.5 %, 5.9 %, and 40.78 % of the variability in sedentary time among preschoolers, respectively. Only the models for FDK, *F*(7,121) = 3.95, *p* < .05, and home-based childcare, *F*(5,14) = 3.61, *p* < .05, were found to be statistically significant.

The unique contribution of each subscale to the prediction of sedentary time within the three environments was explored. For FDK classrooms, Sedentary Environment, Sedentary Opportunities, and Fixed Play Environment were found to predict 25 %, 32 %, and 37 % of the variability, respectively (*p* < .01). Important to note, however, was the inverse relationship identified between Sedentary Opportunities and Fixed Play Environment with the sedentary time of preschoolers in the FDK program. In center-based childcare settings, only Portable Play Environment was significant in explaining approximately 24 % of the variability in sedentary activity (*p* = .05) and the relationship between these variables was positive, while both Sedentary Environment and Sedentary Opportunities approached significance. For home-based childcare, Staff Behaviors was found to account for approximately 54 % of the variability (*p* < .05), and again, the relationship between these variables was positive. Table 2 contains a complete description of the correlations between the EPAO subscales and preschoolers’ sedentary time within the three early learning environments.

**Table 2:** Summary of Coefficients, Confidence Intervals, t-Values, *p*-Values, and Correlations for EPAO Subscales and Sedentary Time

Environment Type	EPAO subscale	B	95 % CI [lower bound, upper bound]	*t*	*p*	Correlations	
						Zero-order	Partial
Home^a*^	Sedentary Opportunities	−.43	[−1.1, .24]	−1.58	.14	−.16	−.39
	Sedentary Environment	.96	[−.36, 2.40]	1.79	.10	−.28	.43
	Portable Play Environment	.12	[−1.95, 2.53]	.23	.82	.59	.06
	Fixed Play Environment	.06	[−1.29, 1.25]	.16	.87	.16	.04
	Staff Behaviors	1.45	[−.17, 2.91]	2.38	.03	.62	.54
Center^b^	Sedentary Opportunities	−.43	[−1.11, .05]	−1.89	.06	−.22	−.23
	Sedentary Environment	.26	[−.14, .63]	1.64	.11	-.03	.20
	Portable Play Environment	.58	[−.37, 1.37]	1.97	.05	.20	.24
	Fixed Play Environment	−.32	[−1.14, .28]	−1.02	.31	−.05	−.13
	Staff Behaviors	−.04	[−.25, .39]	−.44	.66	−.19	−.06
FDK^c*^	Sedentary Opportunities	−.19	[−.32, −.06]	−2.80	.01	−.17	−.25
	Sedentary Environment	.47	[.29, .83]	3.75	.00	.17	.32
	Portable Play Environment	.18	[−.26, .36]	1.21	.23	−.02	.11
	Fixed Play Environment	−.80	[−1.36, −.32]	−3.08	.00	−.10	−.27
	Staff Behaviors	.12	[−.11, .20]	1.68	.10	−.04	.15

*Note*. ^a^Model accounts for 40.7 % of the variability in sedentary time (intercept = 14.35); ^b^Model accounts for 5.9 % of the variability in sedentary time (intercept = 39.86); ^c^Model accounts for 10.5 % of the variability in sedentary time (intercept = 44.97); * = significant model (*p* < .05); CI = confidence interval; EPAO = Environment and Policy Assessment and Observation; FDK = full-day kindergarten

## Discussion

The purpose of this study was to explore sedentary time of preschoolers attending three different early learning environments: FDK, center-, and home-based childcare. Additionally, this research sought to explore the characteristics of these environments which influenced sedentary behaviors.

The primary finding of this work indicated that, in comparison to home-based childcare and FDK programs, preschoolers in center-based childcare accumulated the most sedentary time. In light of recent research recognizing the center-based childcare setting as an obesogenic [24] and sedentary [11, 31] environment, the results of the present study are not surprising. Childcare providers have noted the lack of appropriate *indoor* space [32] and physical activity-specific resources [33] as barriers to engaging preschoolers in gross motor activities and consequently, resulting in increased sedentary behaviors. With regard to *outdoor* play, given that center-based environments tend to be heavily regulated, staff may be inclined to display increased safety concerns for the children’s wellbeing while outdoors, and may therefore limit more vigorous and rambunctious play during care hours (e.g., running, swiftly climbing on jungle gym equipment, etc.) [33]. Moreover, center-based childcare may have less outdoor play space, or portable play equipment compared to FDK schools, which in turn, may increase sedentary behaviors in this environment. However, despite a significant difference in rates of sedentary time, it should be noted that the differences across environments were not large (i.e., preschoolers in center-based settings participated in 0.9 mins/hr and 1.94 mins/hr more than those in home-based childcare and FDK, respectively). This suggests that young children attending all three early learning environments warrant attention and action as the high rates of sedentary time are concerning in light of the associated negative health consequences [3].

In contrast to the above-noted finding, it was found that preschoolers in FDK programs accumulated the least amount of sedentary time. This discovery may be explained by the fact that the participants in this group tended to be at the ‘older’ end of the preschool-aged spectrum (i.e., 4–5 years). As such, these children may have possessed more developed gross motor skills and abilities which might have enabled them to participate in higher intensity activity or more prolonged periods of active play (and less time in sedentary pursuits) [34]. Further, less concentrated supervision during outdoor play periods (or recess) may have also attributed to the finding of decreased sedentary time among this group (i.e., many children of various ages and developmental stages, with fewer teachers/supervisors on-site to monitor activity). Finally, it is possible that this group was less sedentary because these children did not take a nap, where preschoolers in the other two environments would have. Efforts were taken to minimize this difference (e.g., children whose nap was 60 minutes or more would have this data not included for analysis); however, it is still possible that this influenced activity levels. While it is important to note the lowest levels of sedentary time were accumulated by preschoolers in FDK, the fact that this group still spent a significant amount of time in sedentary pursuits (i.e., 39.68 mins/hr) should not be neglected. Similar to the findings of Talley and colleagues which explored physical activity among kindergarten children [35], a large proportion (approximately 66 %) of this group’s day in school was spent being sedentary. Consequently, efforts need to be undertaken by school officials and public health professionals to ensure unnecessary sedentary time be minimized during school hours. Doing so will assist children in developing healthful behaviors relating to physical activity and sedentary time; all of which will hopefully carry forward into later life.

In terms of the environmental characteristics that influence rates of sedentary time among preschoolers across all three early learning environments, many findings warrant comment. First, the subscales Sedentary Environment (positive association), Sedentary Opportunities (negative association), and Fixed Play Equipment (negative association) accounted for a substantial amount of the variation in preschoolers’ sedentary time in FDK programs. Although the link between increased levels of sedentary time and high visibility/prominence of sedentary equipment (e.g., computers, TVs, etc.) in the classroom has been confirmed in the present study and elsewhere [23], the contradictory relationship highlighted was unexpected. Previous research supports that the more access preschoolers have to sedentary activities, the more likely they are to engage in sedentary behaviors [36, 37]. The inverse relationship noted between the presence of fixed equipment (e.g., climbers, jungle gyms) and sedentary levels was also interesting and contradicts the results of the corresponding pilot study [11]. Consequently, there is an ongoing need to both implement and study the impact of strategies meant to minimize sedentary opportunities available to young children during school hours (e.g., limit the availability of screens in the classroom, implement policies that discourage long periods of sitting/inactivity, incorporate physical activity into classroom curriculum).

Within center-based childcare, only the Portable Play Environment subscale was significant, positively predicting close to 25 % of the variability in preschoolers’ sedentary time. This discovery is interesting given the findings of a meta-analysis conducted by Gordon et al. which suggested that portable equipment provides young children with numerous opportunities to move with the equipment and engage in active play [38]. Further, these results are in contrast to the findings of this study’s pilot project (which used the EPAO with a small sample of center-based childcare centres only) which found that portable play equipment had a positive association with preschoolers' physical activity levels in the same environment [11]. One possible explanation for this contradictory finding might be that portable play equipment can be used and manipulated from a seated position (e.g., sitting and throwing a ball). Additionally, it is possible that while portable play equipment is typically associated with increased physical activity levels, in the present study, the centres that participated did not offer adequate space to use the gross motor equipment (e.g., tricycles) as intended.

Lastly, the Staff Behaviors subscale accounted for more than half of the variability in sedentary time among preschoolers in home-based childcare. The importance of childcare providers’ behaviors in this environment has been noted previously [39]. Because a single individual is responsible for caring for all enrolled children in this particular type of setting, it is likely that young children will pay close attention to the childcare provider’s behaviors. Consequently, during care hours, it is important that these key individuals serve as positive role models by engaging in and promoting active behaviors (and discouraging prolonged sedentary time) as well as discussing with children the importance of being active. As such, specific training and educational opportunities for this group may serve as an important resource.

A possible suggestion for curtailing this negative health behavior in all three early learning environments may include increased staff training and education regarding the negative consequences of sedentary time. These environments may also benefit from the introduction of policies which not only articulate required minutes of active play but that also provide specific parameters regarding the minimization of sedentary activities (e.g., prolonged periods of sitting, screen use). One possibility includes pairing previously sedentary activities (e.g., a lesson about the solar system), with movement, making them interactive and engaging for children (e.g., moving around the classroom to the different planets). This will not only decrease their sedentary time, but might also increase their physical activity level.

A limitation of the current study was the use of the EPAO tool to assess the FDK and home-based childcare environments. While no other validated tool exists to explore these facilities, the EPAO was designed for center-based childcare [24, 25]; therefore, it may not have accurately assessed the environmental characteristics within the other two early learning arrangements. Secondly, only those EPAO subscales that have, or were anticipated to, influence sedentary time were included in the analyses. Thirdly, only a small sample of home-based childcare facilities, and consequently preschoolers enrolled in this setting, were successfully recruited. The low participation rate among this type of facility may have limited our ability to make comparisons between preschoolers across the different early learning environment types. Finally, a sedentary behaviors questionnaire was not administered to early learning staff, which might have provided additional contextual data for understanding the types of sedentary activities in which preschoolers engaged (e.g., television viewing, reading) and the importance or reason for these activities (e.g., educational, convenience).

## Conclusion

 Despite the above noted limitations, the present study offers the first exploration of differences in sedentary time among preschoolers in different early learning arrangements. This study also explored the influential attributes of these early learning environments with regard to sedentary pursuits. Findings from this work highlight the ongoing need to reduce sedentary time among preschoolers in early learning programs, particularly within the center-based childcare environment. Interventions focused on minimizing sedentary time and encouraging physical activity within these environments may be an important next step.

## Abbreviations

ANCOVA: Analysis of covariance
EPAO: Environment and policy assessment and observation
FDK: Full-day kindergarten
LEAPP: Learning environments activity potential in preschoolers
